# Gender effect on phenotype and genotype in patients with post-polycythemia vera and post-essential thrombocythemia myelofibrosis: results from the MYSEC project

**DOI:** 10.1038/s41408-018-0128-x

**Published:** 2018-09-21

**Authors:** Daniela Barraco, Barbara Mora, Paola Guglielmelli, Elisa Rumi, Margherita Maffioli, Alessandro Rambaldi, Marianna Caramella, Rami Komrokji, Jason Gotlib, Jean Jacques Kiladjian, Francisco Cervantes, Timothy Devos, Francesca Palandri, Valerio De Stefano, Marco Ruggeri, Richard T. Silver, Giulia Benevolo, Francesco Albano, Michele Merli, Daniela Pietra, Tiziano Barbui, Giada Rotunno, Mario Cazzola, Toni Giorgino, Alessandro Maria Vannucchi, Francesco Passamonti

**Affiliations:** 1 Hematology, Department of Medicine and Surgery, University of Insubria & Ospedale di Circolo, ASST Sette Laghi, Varese, Italy; 2 CRIMM-Centro Ricerca e Innovazione delle Malattie Mieloproliferative, Department of Experimental and Clinical Medicine, Azienda ospedaliera-Universitaria Careggi, University of Florence, Florence, Italy; 3 Department of Hematology Oncology, Fondazione IRCCS Policlinico San Matteo, Università di Pavia, Pavia, Italy; 4 Department of Oncology–Hematology, University of Milan and BMT Unit, ASST Papa Giovanni XXIII, Bergamo, Italy; 5 Ospedale Niguarda Cà Granda, Milano, Italy; 6 Moffit Cancer Center, Tampa, FL USA; 7 Stanford University, Palo Alto, CA USA; 8 Hôpital Saint-Louis et Université Paris Diderot, Paris, France; 9 Hospital Clínic, IDIBAPS, University of Barcelona, Barcelona, Spain; 10 Department of Hematology, University Hospitals Leuven and Laboratory of Experimental Transplantation, Department of Microbiology and Immunology, KU Leuven, Leuven, Belgium; 11 Policlinico S. Orsola-Malpighi, Bologna, Italy; 12 Università Cattolica del Sacro Cuore, Roma, Italy; 13 Ospedale S. Bortolo, Vicenza, Italy; 14 Weill Cornell Medical College, New York, NY USA; 15 SC Hematology, A.O. Città della Salute e della Scienza, Turin, Italy; 16 Università di Bari, Bari, Italy; 17 Research Foundation, ASST Papa Giovanni XXIII, Bergamo, Italy; 18 Biophysics Institute, National Research Council of Italy, c/o Department of Biosciences, University of Milan, via Celoria 26, I-20133 Milan, Italy

Myeloproliferative neoplasms (MPN) include essential thrombocythemia (ET), polycythemia vera (PV), and primary myelofibrosis (PMF) and are established clonal disorders ^1^. MPN diagnosis affects survival of individuals as compared with matched populations. Concerning PMF, survival is currently stratified on the basis of the International Prognostic Scoring System (IPSS)^2^ and its variants or on the most recent mutation-based MIPSS-70^3^. In post-PV MF and post-ET MF, namely secondary myelofibrosis (SMF), the MYSEC PM (Myelofibrosis Secondary to PV and ET-Prognostic Model), has been recently developed to assess survival^4^. Epidemiological data have revealed an advantage for women in surviving a diagnosis of cancer compared with men^5^. Sex hormones might play a role in the hematopoiesis and in the pathogenesis of hematologic malignancies^6^. Concerning MPNs, gender differences have been observed in terms of disease distribution (higher prevalence of females in ET and of males in PV), *JAK2V617F* allele burden (lower in females)^7^, and numbers of homozygous mutant colonies (larger in males)^8^.

In this study, we assessed the prognostic impact of gender in the study population of the multicenter MYSEC project including 684 SMF patients with driver mutational status available. Diagnosis of SMF was performed between 1981 and 2015 and were locally reviewed according to the International Working Group on Myeloproliferative Neoplasm Research and Treatment criteria (IWG-MRT 2008)^1^. Molecular and genetic tests were performed as previously described^9,10^. The study was approved by the ethical committee of each institution and conducted in accordance with the Declaration of Helsinki. Statistical analyses considered clinical and laboratory data collected at the time of progression to SMF. Wilcoxon rank sum and Pearson’s chi-squared tests were performed to report differences between the groups, whereas Kaplan–Meier estimators, log-rank tests, and Cox regression models were used for time-to-event analysis.

The first observation we found is that diagnosis of ET and PV occurred at younger age in females versus males (median, 50 vs. 53 years, *p* = 0.027) and that age at the time of SMF transformation was similar between the genders (median 63 vs. 65 years, *p* = 0.23). This is in favor of a slower progression of the diseases in females, as documented by a longer time to progression in SMF (median 11.3 vs. 10.1 years, *p* = 0.015).

Table 1 outlines clinical and laboratory features at diagnosis of SMF, stratified by gender. Overall, 328 patients (48%) were females and the female/male ratio was 0.92. The cohort consisted of 332 PET MF and 352 PPV MF, of which 167 (50%) and 161 (46%) were females, respectively (*p* = 0.23). In SMF, female sex was correlated with higher platelet count (*p* = 0.041), smaller palpable spleen (*p* = 0.016), and lower frequency of circulating blasts ≥ 1% (*p* = 0.008). We found also a correlation with lower hemoglobin levels (*p* = 0.036), but this was evident only in younger women as a possible expression of a pre-menopausal phase. Within the PET MF cohort, female sex was significantly associated with smaller palpable spleen (*p* = 0.024) and lower frequency of circulating blasts ≥1% (*p* = 0.027). Conversely, we did not find gender differences within PPV MF cohort.

**Table 1:** Presenting clinical and laboratory features of 684 patients with secondary myelofibrosis, stratified by gender

	SMF			PET-MF			PPV-MF		
	Female	Male	*P*	Female	Male	*P*	Female	Male	*P*
Median age at diagnosis of SMF	63 (30–96)	65 (25–89)	0.23	62 (30–93)	64 (25–84)	0.36	64 (34–96)	66 (38–89)	0.55
Median follow up, years (95% CI)	2. 9 (0–27)	3 (0–19)	0.76	2.9 (0–14)	3.2 (0–17)	0.53	3.2 (0–27)	2.9 (0–19)	0.31
WBC median (range), x10^9^/L	10 (1.7–97.3)	10.4 (1.1–98.4)	0.89	8 (1.9–97.3)	7.5 (1.1–86)	0.15	12.4 (1.7–88.7)	14 (3–98.4)	0.68
Hb median (range), x10^9^/L	10.9 (5–15.7)	11.6 (5.4–15.7)	**0.036**	10.6 (5–15.6)	10.9 (5.4–15.7)	0.45	11.5 (7.4–15.7)	12.1 (6.8–15.6)	0.12
Hb, category^a^, *n* (%)			**0.001**			**0.014**			**0.001**
Severe	11 (4)	23 (7)		8 (5)	19 (12)		3 (2)	4 (2)	
Moderate	84 (27)	63 (19)		53 (32)	40 (25)		31 (21)	23 (13)	
Mild	117 (38)	188 (56)		71 (43)	84 (52)		46 (31)	104 (60)	
Normal	97 (31)	60 (18)		31 (19)	17 (11)		66 (45)	43 (25)	
Over	2 (1)	0 (0)		1 (1)	0 (0)		1 (1)	0 (0)	
PLT median (range), x10^9^/L	356 (15–1908)	315 (16–1418)	0.**041**	418 (51–1908)	352 (40–1213)	**0.075**	302 (15–1689)	278 (16–418)	0.49
Presence of circulating blast, *n* (%)	65 (22)	102 (32)	**0.008**	29 (19)	47 (32)	**0.01**	36 (26)	55 (32)	0.24
Spleen^b^, median (range)	6 (0–34)	8 (0–27)	**0.0016**	4 (0–25)	5 (0–27)	**0.024**	8 (0–34)	10 (0–27)	0.57
Constitutional symptoms, *n* (%)	135 (44)	150 (45)	0.73	57 (37)	56 (36)	0.97	78 (50)	94 (52)	0.77
Normal karyotype^c^, *n* (%)	101 (64)	122 (68)	0.41	58 (70)	60 (77)	0.31	43 (57)	62 (61)	0.57
Favorable karyotype^d^, *n* (%)	131 (85)	154 (87)	0.52	117 (70)	114 (69)	0.85	63 (85)	83 (83)	0.7
Driver mutational status, *n* (%)									
* JAK2*	257 (79)	276 (78)	0.8	96 (58)	85 (52)	0.28	161 (100)	191 (100)	1
* CALR*	47 (14)	55 (15)	0.68	47 (28)	55 (33)	0.3			
* MPL*	17 (5)	13 (4)	0.33	17 (10)	13 (8)	0.47			
Triple negative	7 (2)	12 (3)	0.33	7 (4)	12 (7)	0.23			
Time between ET/PV and SMF (years)	11.3 (0–39)	10.1 (0–41)	**0.0015**	11.4 (0.4–35)	9.7 (0.3–30)	**0.005**	11.1 (0–39)	10.8 (0.4–41)	0.49
Thrombotic events post SMF, *n* (%)	37 (11)	30 (8)	0.21	18 (11)	11 (7)	0.18	19 (12)	19 (10)	0.59
Leukemic transformation, *n* (%)	25 (8)	27 (8)	0.98	15 (9)	15 (9)	0.97	10 (6)	12 (6)	0.98
Deaths, *n* (%)	66 (20)	102 (29)	**0.028**	26 (16)	43 (26)	**0.037**	40 (25)	59(31)	0.45

*ET* essential thrombocythemia, *PV* polycythemia vera, *SMF* secondary myelofibrosis, *PET MF* post-essential thrombocythemia myelofibrosis, *PPV MF* post-polycythemia vera myelofibrosis, *WBC* white blood cell count, *Hb* hemoglobin level, *PLT* platelet count

^a^Nicolosi et al.^14^

^b^Palpable from the left costal margin

^c^Cytogenetic information available in 339 patients

^d^Favorable karyotype: normal karyotype or sole or two abnormalities that do not include the unfavorable cytogenetic abnormalities (complex karyotype or sole or two abnormalities that include +8, −7/7q-, i(17q), −5/5q-, 12p-, inv(3), or 11q23 rearrangement)

Bold font indicates significant *p*–values

Driver mutational status within SMF females was *JAK2* in 257 (78%), *CALR* in 47 (14%), *MPL* in 17 (5%), and triple negative in seven females (TN, 2%). Within females with PET MF, the distribution was as follows: 96 *JAK2* (57%), 47 *CALR* (28%), 17 *MPL* (10%), 7 TN (4%). Mutation frequency was similar in the two genders (*p* = 0.57). Furthermore, female gender frequency per genotype was 46% in *JAK2-*pos PPV MF, 53% in *JAK2*-pos PET MF, 46% in *CALR*-pos, 57% in *MPL*-pos, and 37% in TN cases without significant differences with respect to male gender (*p* = 0.9). Concerning cytogenetic profile, abnormalities have been described in 58 females and 58 males (36 vs. 32%, *P* = 0.41). Of interest, the rate of complex karyotype (non monosomal) was lower in females versus males (3 vs. 12, i.e. 2 vs. 7%, *p* = 0.03).

During a median follow-up of 3 years (range, 0.6–27.3), 67 (10%) thrombosis occurred with an incidence of 3.1/100 patient-years and 2.4/100 patient-years in the female and male groups, respectively without significant differences (*p* = 0.32). In addition, the rate of fatal thrombosis was superimposable. Concerning blast phase (BP), 52 (8%) transformations occurred with an incidence of 1.8/100 patient-years and 2/100 patient-years in the female and male groups, respectively (*p* = 0.88).

Death occurred in 168 (25%) patients. Overall survival from diagnosis of SMF was better in female (median values, 10.1 years, 95% CI: 8.1-NR) than in male patients (8.1 years, 95% CI: 6.8-NR), with a hazard ratio (HR) of 0.68 (95% CI: 0.50–0.92; log rank test *p* = 0.013, Fig. 1). The correlation that we found, remained statistically significant even after adjusting for age at SMF diagnosis (HR 0.71, 95% CI: 0.52–0.97; *p* = 0.03) with a Cox regression model, was uninfluential (*p* = 0.3). Of note, female gender retained a survival advantage, albeit with a weak statistical significance, even when added as a covariate to the MYSEC-PM risk strata and type of diagnosis (PPV MF and PET MF) (HR 0.72, 95% CI: 0.51–1.02; *p* = 0.06). Taking into account earlier disease onset in females, a multivariable analysis including age at diagnosis and gender found that female sex still remains significant (HR: 0.71, 95% CI: 0.52–0.97; *p* = 0.03).

**Fig. 1:**
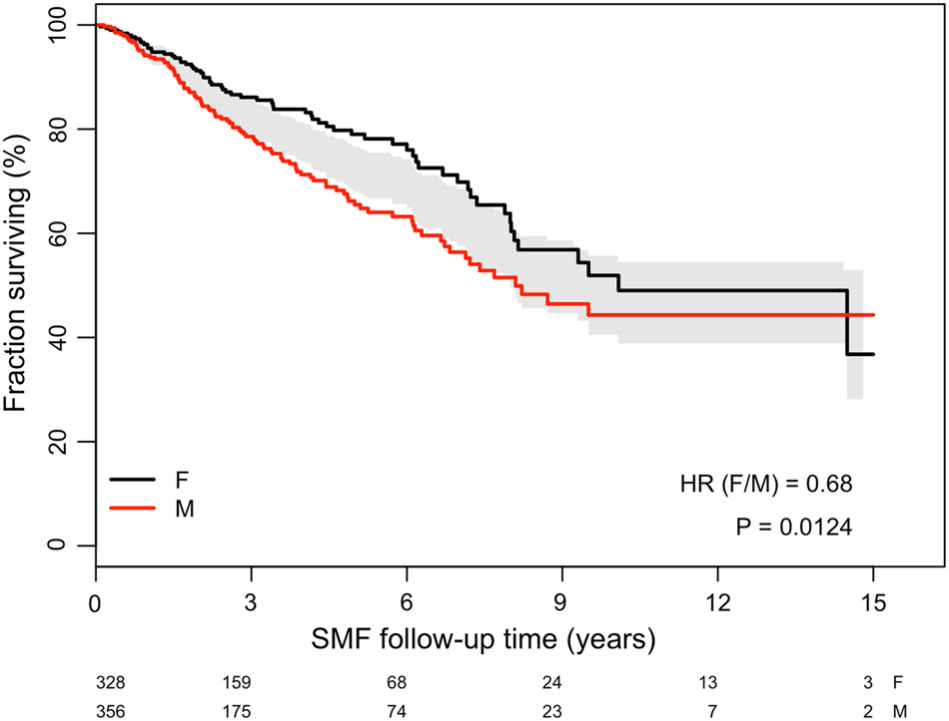
**Impact of gender on overall survival in 684 patients with post-polycythemia vera and post-essential thrombocythemia myelofibrosis**

The current study indicates that women are characterized by a slower progression from PV/ET to SMF, more indolent phenotype at SMF (higher platelet count, lesser degree of splenomegaly and of circulating blasts) and a longer life expectancy than male counterpart. Survival difference between men and women in the general population is well recognized, and in cancer subset shorter survival might be linked to host factors, such as activity of sex steroid hormone pathway, occupational exposures, and lifestyle factors, as already described^5^.

From a biological viewpoint, MPNs patients exhibit decreased telomere length, more evident in men than in women^11^. This is indicative of a lower genomic instability in females during disease progression. Looking for gene expression between genders in MPNs, a differential gene expression in female patients as compared with male patients has been reported with 235 genes differentially regulated in women, vs. 571 genes differentially regulated in men^12^. In our analysis, we found that women have a lower rate of complex karyotype (non monosomal) as a result of lesser genomic instability. The correlation between complex karyotype and worse survival has been recently documented in SMF^10^. This could add explanations to the different survivals that we found between the genders.

The impact of gender on clinical phenotype has been recently investigated in ET and PMF, but never in SMF. Tefferi et al.^13^ demonstrated that older women with ET live longer than their male counterparts and that gender might supersede thrombosis history as a risk variable for overall survival. An analysis on 1109 PMF showed that mild anemia, defined as hemoglobin between 10 g/dl and sex-adjusted lower limit of normal, independently predicted shortened survival in men, but not in women^14^.

The magnitude of difference in benefit we found in term of survival between genders (average HR of 0.68, meaning 32% reduction of the risk of death for women with respect to men) seems clinically relevant and has some implications. The most practical is for the doctor/patient communication at the time of diagnosis of SMF. The second is for helping decision making in those areas where a case by case decision is suggested. So far, new therapeutic approaches in MPNs have not taken sex into consideration. However, focusing on subgroup analysis of the COMFORT trials, women receiving ruxolitinib have better survival than men (HR: 0.70; 95% CI: 0.49–0.998)^15^, this can eventually overbalance the overall drug results.

In conclusion, our study in SMF indicates that female gender has a more indolent disease with a specific phenotype and better prognosis. This finding finally led to a more accurate definition of the natural history of the disease and drove the attention toward a careful interpretation of clinical trial results.

## References

[1] Passamonti F, Maffioli M (2016). Update from the latest WHO classification of MPNs: a user’s manual. Hematol. Am. Soc. Hematol. Educ. Program.

[2] Cervantes F (2009). New prognostic scoring system for primary myelofibrosis based on a study of the international working group for myelofibrosis research and treatment. Blood.

[3] Guglielmelli P (2018). MIPSS70: mutation-enhanced international prognostic score system for transplantation-age patients with primary myelofibrosis. J. Clin. Oncol..

[4] Passamonti F (2017). A clinical-molecular prognostic model to predict survival in patients with post polycythemia vera and post essential thrombocythemia myelofibrosis. Leukemia.

[5] Srour SA (2016). Incidence and patient survival of myeloproliferative neoplasms and myelodysplastic/myeloproliferative neoplasms in the United States, 2001-12. Br. J. Haematol..

[6] Ratajczak MZ (2017). Why are hematopoietic stem cells so ‘sexy’? on a search for developmental explanation. Leukemia.

[7] Stein BL (2010). Sex differences in the JAK2 V617F allele burden in chronic myeloproliferative disorders. Haematologica.

[8] Godfrey AL (2013). Clonal analyses reveal associations of JAK2V617F homozygosity with hematologic features, age and gender in polycythemia vera and essential thrombocythemia. Haematologica.

[9] Passamonti F (2017). Driver mutations’ effect in secondary myelofibrosis: an international multicenter study based on 781 patients. Leukemia.

[10] Mora B (2018). Value of cytogenetic abnormalities in post-polycythemia vera and post-essential thrombocythemia myelofibrosis: a study of the MYSEC project. Haematologica.

[11] Bernard L (2009). Telomere length is severely and similarly reduced in JAK2V617F-positive and -negative myeloproliferative neoplasms. Leukemia.

[12] Spivak JL (2014). Two clinical phenotypes in polycythemia vera. N. Engl. J. Med.

[13] Tefferi A (2017). Gender and survival in essential thrombocythemia: A two-center study of 1,494 patients. Am. J. Hematol..

[14] Nicolosi M (2018). Sex and degree of severity influence the prognostic impact of anemia in primary myelofibrosis: analysis based on 1109 consecutive patients. Leukemia.

[15] Vannucchi AM (2015). A pooled analysis of overall survival in COMFORT-I and COMFORT-II, 2 randomized phase III trials of ruxolitinib for the treatment of myelofibrosis. Haematologica.

